# Child sexual abuse in India: A systematic review

**DOI:** 10.1371/journal.pone.0205086

**Published:** 2018-10-09

**Authors:** Vikas Choudhry, Radhika Dayal, Divya Pillai, Ameeta S. Kalokhe, Klaus Beier, Vikram Patel

**Affiliations:** 1 Public Health Foundation of India, Institutional Area, Gurugram, Haryana, India; 2 Sambodhi Research and Communications Pvt. Ltd., Noida, Uttar Pradesh, India; 3 Emory University School of Medicine, Department of Medicine, Division of Infectious Diseases, Atlanta, Georgia, United States of America; 4 Emory University Rollins School of Public Health, Department of Global Health, Atlanta, Georgia, United States of America; 5 Institute of Sexology and Sexual Medicine, Charité - Universitätsmedizin Berlin, Corporate member of Freie Universität Berlin, Humboldt-Universität zu Berlin, and Berlin Institute of Health, Luisenstraße, Berlin, Germany; 6 Department of Global Health and Social Medicine, Harvard Medical School, Boston, MA, United States of America; Massachusetts General Hospital, UNITED STATES

## Abstract

**Objective:**

Child Sexual Abuse (CSA) is a pressing human right issue and public health concern. We conducted a systematic review of quantitative and qualitative studies published in the past decade on CSA in India to examine the distribution of the prevalence estimates for both genders, to improve understanding of the determinants and consequences of CSA and identify gaps in the current state of research.

**Methods:**

For this systematic review, we searched electronic literature databases (PubMed, POPLINE, and PsycINFO) for articles published in English on Child Sexual Abuse in India between January 1, 2006 and January 1, 2016 using 55 search terms. Data were extracted from published articles only.

**Findings:**

Fifty-one studies met inclusion criteria for the review. The review indicates that prevalence rates of CSA is high among both boys and girls in India. Due to heterogeneity of study designs and lack of standardised assessments, reported prevalence estimates varied greatly among both genders in different studies. There is a need to conduct representative studies using a validated instrument to obtain valid epidemiological estimates. Commercial sex workers, men who have sex with men, and women with psychiatric disorders were at higher risks for sexual abuse during childhood. In addition, the synthesis of qualitative data across studies included in the review suggests that exposure and perpetration of CSA is a multifaceted phenomenon grounded in the interplay between individual, family, community, and societal factors. The review indicates poor physical, behavioural, social, and mental health outcomes of CSA in India. We conclude with a research agenda calling for quantitative and qualitative studies to explore the determinants and perpetration of child sexual abuse in India from an ecological lens. This research agenda may be necessary to inform the development of a culturally tailored primary prevention and treatment strategy for CSA victims in India.

## Introduction

The World Health Organization (WHO) defines Child Sexual Abuse (CSA) as “*the involvement of a child in sexual activity that he or she does not fully comprehend*, *is unable to give informed consent to*, *or for which the child is not developmentally prepared and cannot give consent*, *or that violates the laws or social taboos of society…*”[1]. CSA includes an array of sexual activities like fondling, inviting a child to touch or be touched sexually, intercourse, exhibitionism, involving a child in prostitution or pornography, or online child luring by cyber-predators [2, 3].

CSA is a serious problem of considerable magnitude throughout the world. A recent systematic review of 55 studies from 24 countries found much heterogeneity in studies in terms of definition and measurement of CSA and concluded that rates of CSA ranged from 8 to 31% for females and from 3 to 17% for males [4]. Despite similar methodological challenges, other systematic reviews which included studies conducted worldwide across hundreds of different age-cohort samples have observed alarming rates of CSA, with averages of 18–20% for females and of 8–10% for males, with the lowest rates for both girls (11.3%) and boys (4.1%) found in Asia, and highest rates found for girls in Australia (21.5%) and for boys in Africa (19.3%) [5, 6].

CSA has profound consequences for the child. It is known to interfere with growth and development [7, 8]. CSA has also been linked to numerous maladaptive health behaviors, and poor social, mental and physical health outcomes throughout the lifespan [2, 9–11]. In accordance with that, there is evidence that CSA can affect neuro-biological systems, e.g. the cortical representation of the genital somatosensory field [12]. Other common sequelae for adult survivors of CSA may include relational challenges (e.g., increased risk for domestic violence), violent behaviors, and increased risk of perpetration of CSA as adults [2, 13].

Children, under the age of 18, contribute to 37% of India's population [14] with large proportions experiencing great deprivations such as lack of access to basic education, nutrition or health care [15]. In addition, they are susceptible to different forms of adverse childhood experiences (ACEs) including various forms of abuse, neglect, and maltreatment with child protection remaining largely unaddressed [16–18]. A large-scale national study conducted in 2007 by Ministry of Women and Child Development (MoWCD), to assess the extent and nature of child abuse in India, uncovered some alarming statistics; that among the 12,447 children interviewed, more than half (53 percent) reported experience of sexual abuse, defined as “sexual assault, making the child fondle private parts, making the child exhibit private body parts and being photographed in the nude” and over 20 percent reported severe sexual abuse [19]. While these statistics need to be interpreted with caution as it was conducted in a convenience rather than nationally representative sample, the numbers speak to the significance of the problem and highlight particularly high-risk groups. Smaller studies from India have also reported very high prevalence of CSA [17, 20, 21].

Increased attention in the public discourse and activism around child protection led to the Government of India passing the, ‘The Protection of Children from Sexual Offences (POCSO)’ law in 2012. This act criminalizes a range of acts including rape, harassment, and exploitation for pornography involving a child below 18 years of age and mandates the setting up of Special Courts to expedite trials of these offences. However, the issue of CSA remains a taboo in India. As around the world, the research findings in India support significant underreporting of CSA to authorities versus reporting in protected research settings [17, 22]. Only 3% of CSA offences uncovered by national level study in 2007 were reported to the authorities. Renuka Chowdhury, the then minister of women and child development, in her introduction to this national survey report of MoWCD referred CSA as “…shrouded in secrecy with a conspiracy of silence around the entire subject” [19].

A systematic review of prevalence estimates, determinants, and impact of CSA are important for the development of prevention programs and the provision of support. Even though some literature on CSA, from India, has been published as an epidemiological overview and narrative reviews, and some empirical studies have been included in the international literature, there is no systematic review of the literature on CSA in India. This paper presents a systematic review of a wide range of studies, both quantitative and qualitative, conducted over the past decade on CSA in India. It aims to examine the distribution of the prevalence of CSA estimates for both genders, improve understanding of the determinants and consequences of CSA, and identify gaps in the current state of research.

## Methods

The research questions, inclusion criteria, search strategy, search terms, search engines, and study protocol for the proposed study were developed in consultation with a panel of experts who have been working in the field of sexual violence in India (names included in Acknowledgments) and along the guidelines as per the Preferred Reporting Items for Systematic Reviews and Meta-Analyses (PRISMA) [23] (S1 Checklist). We searched electronic literature databases (PubMed, POPLINE, and PsycINFO) to search for articles published on CSA in India. Searches were conducted using both medical subject headings (MeSH) and keywords. An example for the use of search term like sexual assault among 55 other terms (Table 1) that was used is *“…*.*(("sexual assault"[MeSH Terms] OR ("sexual"[All Fields] AND "assault"[All Fields]) OR "sexual assault"[All Fields] OR ("india"[MeSH Terms] OR "india"[All Fields])” OR ((“sex offenses"[MeSH Terms] OR ("sex"[All Fields] AND "offenses"[All Fields]) OR "sex offenses"[All Fields]” AND ("india"[MeSH Terms] OR "india"[All Fields])* across the three databases. The same search strategy was used across all three databases.

**Table 1:** Search terms paired with “India”.

**Sexual violence**	**Rape**	**Sexual Violence Survivor**	**Adult survivors of child adverse event**	**Gender based violence**
Sexual abuse	Prostitution	Child abuse survivor	Psychological sexual dysfunction	Human trafficking
Sexual assault	Crime victims	Sexual offender	Female genital mutilation	Intimate partner violence
Sexual coercion	Incest	Paedophilia	Domestic violence	Emotional violence
Sexual aggression	Perpetrator	Sexual abuse dysfunction	Sexual maltreatment	Sexually harmful behaviour
Sexual offense	Paedophile	Pornography	Paraphilic disorder	Sexual exploitation
Sexual victim	Sodomy	Non-consensual sex	Cyber sexual crime	Sexual harassment
Transvestism	Dating violence	Marital rape	Sexual deviance	Atypical sexual behaviour
Exhibitionism	Physical violence	Abusive images	Juvenile delinquency	Battered child syndrome
Voyeurism	Masochism	Molestation	Exposure to violence	Effects of violence
Fetishism	Stalking	Sexual crime	Hebephilia	Online sexual offender

### Study selection

We included studies that were published in English between January 1, 2006 and January 1, 2016, involved human subjects, and collected primary data on experience, perpetration, or response to CSA in India. Grey literature, reports, studies not collecting or analyzing original CSA data (i.e. epidemiological overview, meta-analyses, systematic reviews), commentaries, and editorials were not included in the study. The study period was chosen to report results from the recent literature on prevalence, correlates, and consequences of CSA in Indian context.

We included observational studies (*e*.*g*. cross-sectional studies, cohort studies, and case-control studies) that examined prevalence and incidence of CSA and/or associations of CSA with other independent or dependent variables. The qualitative studies that examined the experiences of respondents that had been victims of CSA were also included. Articles examining sexual abuse experience at ≤ 18 years were included. The review also included 1) articles in which CSA data were collected retrospectively from adults and 2) articles which use age bands which cross the 18-years cut-point (i.e. they collect aggregate sexual violence data for youth aged 10–19, 15–24 etc.). In the latter scenario, authors were contacted for disaggregate data on CSA for ages ≤ 18 years. CSA perpetration studies were also included if they measured perpetration of sexual violence against an individual ≤ 18 year of age (irrespective of the age of the perpetrator). Finally, interventions to prevent and/or treat CSA including randomised control trials were also included in this review.

The peer reviewed articles identified from the electronic databases were transferred and stored in EndNote software X7.5. The relevancy check for all the articles at the step of title and abstract screening was conducted by one of the authors (DP or RD) designated as primary reviewers. In addition, the secondary reviewers (VC or AK) conducted relevancy check for 10% of the randomly selected articles at this step along with relevancy check for all the articles that were deemed as “cannot determine” by the primary reviewers. The primary and secondary reviewers conducted relevancy checks in teams that constituted of one primary and one secondary reviewer for the articles that were selected for full text screening (n = 965). The discrepancies on relevancy of the articles between primary and secondary reviewer were noted and discussed by the entire team in a monthly meeting.

### Data extraction and quality assessment

The eligible articles went through a standardized data extraction and quality assessment process. The data extraction form was refined during the extraction of the first few articles to ensure that the forms were comprehensive. The primary reviewers extracted the relevant data in MS Excel for each of the articles that were deemed suitable for inclusion. A few studies were based on same datasets, but we decided to include these studies as different if they assessed different forms of CSA or measured different outcomes associated with CSA. We extracted descriptive characteristics of the sample (e.g. publication date, age of the sample, gender, etc.) from each quantitative (Tables 2–8) and qualitative study (Tables 9 and 10). As contextual moderators, we extracted data on the setting (i.e. schools, colleges, community, shelter homes), and data collection methods (face-to-face interview, focus group discussions or anonymous self-reports). Data on perpetration was also extracted and reported depending on the information available.

**Table 2:** Characteristics of quantitative and mixed-method studies included in the review with school/college, clinical, and community sample.

Author	Period of prevalence	Study design	Setting	Method of data collection	Instruments/ Tools used to measure CSA	Sample size and sampling strategy	Age (in years)	Type of sexual abuse			Prevalence	Perpetration
								Non-contact	Contact	Forced intercourse		
Charak et al. (2015)^1^ [70]	Childhood	Cross-sectional	Schools	Administered structured interviews	Childhood Trauma Questionnaire	291 girls 411 boys *Stratified random sampling*	13–17	X	X	X	48% (35%girls; 57%boys)	Not included
Charak et al. (2014)^1^ [47]	Childhood	Cross-sectional	Schools	Administered structured interviews	Childhood Trauma Questionnaire	294 girls 408 boys *Stratified random sampling*	13–17	X	X	X	48% (35%girls; 57%boys)	Not included
Sahay et. al (2013) [96]	Childhood	Case-control (41 Case and 164 controls)	Schools	Administered structured interviews	Not stated	75 girls 130 boys *Purposive sampling*	12–19	*Not specified*: *“sexual abuse”*			4%	Not included
Miller et al. (2014)^#^ [57]	Childhood	Cross-sectional	Schools	Administered structured interviews	Sexual violence perpetration	309 boys *Stratified Random sampling*	10–16		X	X	10.06%	Included
Hasnain et al. (2006) [97]	Childhood	Cross-sectional	College	Administered structured interviews	Biographical inventory developed with CSV questions	150 girls *Convenience sampling*	>18	X	X	X	38%	Not reported
Das et al. (2014) ^#^ [54]	Childhood	Cross-sectional	Schools	Administered structured interviews	Not stated	1040 boys *Stratified random samplin*g	10–16	X	X	X	Not given	Included
Zolotor et al. (2009) [29]	Past year experience of CSA	Cross Sectional	School	Administered structured interviews	ISPCAN Child Abuse Screening Tool Children's Version (ICAST-C)	53 girls 69 boys *Convenience sampling*	12–17	X	X	X	20%- At Home 8%- At school or any institution	Not reported

* Mixed Methods Study.

**International Society for the Prevention of Child Sexual Abuse and Neglect Tool-Retrospective (ISPCAN).

*** Ministry of Women and Child Development (MoWCD).

^1^ Studies based on same data set but reporting different covariates and outcomes of CSA.

^2^ Studies based on same data set but reporting different outcomes associated with CSA.

# Data based on an Intervention Study.

## Data based on the same Intervention Study. The study was conducted with respondents between 16–24 years of age, but the analysis was restricted to respondents between 16–18 years of age for the purpose of this review.

**Table 3:** Characteristics of quantitative and mixed-method studies included in the review with school/college, clinical, and community sample.

Author	Period of prevalence	Study design	Setting	Method of data collection	Instruments/ Tools used to measure CSA	Sample size and sampling strategy	Age (in years)	Type of sexual abuse			Prevalence	Perpetration
								Non-contact	Contact	Forced intercourse		
Deb et al. (2012)^2^ [48]	Childhood	Cross-sectional	Schools	Administered structured interview	ISPCAN** Child Abuse Screening Tool Children’s Version (ICAST-C)	160 girls 160 boys *Multi-stage random sampling*	14–19	X	X	X	18% (25%girls; 11%boys)	Not reported
Deb et al. (2010)^2^ [50]	Childhood	Cross-sectional	Schools	Administered structured interview	ISPCAN** Child Abuse Screening Tool Children’s Version (ICAST-C)	160 girls 160 boys *Multi-stage random sampling*	14–19	X	X	X	18% (25%girls; 11%boys)	Not reported
Jaisoorya et al. (2015) [67]	Childhood	Cross-sectional	Schools	Administered structured questionnaire	4 questions on experience of sexual abuse	3640 girls 3740 boys *Stratified random sampling*	12–18	X	X	X	4–9%- Non-OCD adolescents 24–41%- OCD adolescents	Not included
Krishnakumar et al. (2014) [32]	Childhood	Cross-sectional	Schools	Self-administered questionnaire	Adapted MoWCD*** questionnaire on child abuse	926 girls 688 boys *Stratified random sampling*	15–19	X	X	X	35% (35%girls; 36%boys)	Not included
Bhilwar et al. (2015) [46]	Childhood	Cross-sectional	Schools	Self-administered questionnaire	Adapted MoWCD*** questionnaire on child abuse	520 girls 416 boys *Stratified random sampling*	18–25	X	X	X	3.0%-14% (3–18%girls; 1–27% boys)	Not included
Jaya et al. (2007) [49]	Childhood	Cross-sectional	Community	Face-to-face interviews, audio computer assisted self-interviews, and interactive interviews	Not stated	474 Girls 583 Boys *Simple random sampling*	15–17		X	X	4.0–41% girls; 12–27% boys	Included

* Mixed Methods Study

**International Society for the Prevention of Child Sexual Abuse and Neglect Tool-Retrospective (ISPCAN)

*** Ministry of Women and Child Development (MoWCD)

^1^ Studies based on same data set but reporting different covariates and outcomes of CSA

^2^ Studies based on same data set but reporting different outcomes associated with CSA

# Data based on an Intervention Study

## Data based on the same Intervention Study. The study was conducted with respondents between 16–24 years of age, but the analysis was restricted to respondents between 16–18 years of age for the purpose of this review

**Table 4:** Characteristics of quantitative and mixed-method studies included in the review with school/college, clinical, and community sample.

Author	Period of prevalence	Study design	Setting	Method of data collection	Instruments/ Tools used to measure CSA	Sample size and sampling strategy	Age (in years)	Type of sexual abuse			Prevalence	Perpetration
								Non-contact	Contact	Forced intercourse		
Nayak et al. (2010) [71]	Childhood	Cross- sectional	Community	Administered structured interviews	2 questions on childhood sexual victimization	1137 men *Stratified random sampling*	16–49	*Not specified*: *“do or watch sexual things”*			9%	Not included
Dunne et al. (2009) [28]	Childhood	Cross-sectional	Community	Administered structured interview	ISPCAN Child Abuse Screening Tools Retrospective version (ICAST-R)	124 young adults (sex disaggregated data not available) *Convenience sampling*	18–26	X	X	X	3–25%	Included
Pillai et al. (2008) [33]	Past year experience of CSA	Cross sectional	Community	Administered structured interviews	Non-standardized questionnaire	1017 girls 1031 boys *Cluster sampling*	12–16	*Not specified*: *“sexual abuse*			3% (4%girls; 2%boys)	Not included
Bhattacharya et al. (2012) [53]	Childhood	Retrospective review of case notes	Medical Records	Case notes abstracted	Clinical case history	52 females *Purposive sampling*	Not specified			X	71.10%	Included
Kar et al. (2007) [56]	Childhood	Cross Sectional	Community	Postal questionnaire	Sexual functioning questionnaire	33 males 28 females *Convenience sampling*	20–58				Not given	Included
Sahay et al. (2010) * [62]	Childhood	Cross-sectional	Community	Self-administered questionnaire	Non-standardized questionnaire	350 women (tribal and non-tribal) *Purposive sampling*	10–18	X	X		41.2% Tribal; 46.6%—Non- tribal	Not included
Sahay et al. (2013) * [59]	Childhood	Cross-sectional	Schools	Self-administered questionnaire	Not stated	110 girls 20 boys *Stratified Random sampling*	8–30	X	X		6.3–38% (8% girls; 10–55% boys)	Not included

* Mixed Methods Study

**International Society for the Prevention of Child Sexual Abuse and Neglect Tool-Retrospective (ISPCAN).

*** Ministry of Women and Child Development (MoWCD).

^1^ Studies based on same data set but reporting different covariates and outcomes of CSA.

^2^ Studies based on same data set but reporting different outcomes associated with CSA.

# Data based on an Intervention Study.

## Data based on the same Intervention Study. The study was conducted with respondents between 16–24 years of age, but the analysis was restricted to respondents between 16–18 years of age for the purpose of this review.

**Table 5:** Characteristics of quantitative and mixed-method studies included in the review with school/college, clinical, and community sample.

Author	Period of prevalence	Study design	Setting	Method of data collection	Instruments/ Tools used to measure CSA	Sample size and sampling strategy	Age (in years)	Type of sexual abuse			Prevalence	Perpetration
								Non-contact	Contact	Forced intercourse		
Pillai et al. (2009) ^##^ [64]	Childhood	Cross-sectional (Baseline data for a Exploratory controlled evaluation for an intervention)	Community (Two Rural and Two Urban)	Administered structured interviews	Non-standardized questionnaire	765 girls and 725 boys *Purposive sampling*	16–18	X	X	X	15.7% girls: 21.2% boys	Not included
Balaji et al. (2011) ^##^ [75]	Childhood	Cross-sectional (End line data for a Exploratory controlled evaluation for an intervention)	Community (Two Rural and Two Urban)	Administered structured interviews	Non-standardized questionnaire	735 girls and 684 boys *Purposive sampling*	16–18	X	X	X	6.7% girls: 11.1% boys	Not included

* Mixed Methods Study.

**International Society for the Prevention of Child Sexual Abuse and Neglect Tool-Retrospective (ISPCAN).

*** Ministry of Women and Child Development (MoWCD).

^1^ Studies based on same data set but reporting different covariates and outcomes of CSA.

^2^ Studies based on same data set but reporting different outcomes associated with CSA.

# Data based on an Intervention Study.

## Data based on the same Intervention Study. The study was conducted with respondents between 16–24 years of age, but the analysis was restricted to respondents between 16–18 years of age for the purpose of this review.

**Table 6:** Characteristics of quantitative and mixed-method studies included in the review with populations at risk.

Author	Period of prevalence	Study design	Setting	Method of data collection	Instruments/ Tools used to measure CSA	Sample size and sampling strategy	Age (in years)	Type of sexual abuse			Prevalence	Perpetration
								Non-contact	Contact	Forced intercourse		
Silverman et al. (2007) [60]	Childhood	Review of sex trafficked females	NGO	Case and medical records abstracted	Not stated	160 Sex trafficked girls and women *Purposive sampling*	<18	X	X	X	51.87%	Included
Silverman et al. (2006) [72]	Childhood	Case and medical records	NGO	Case and medical records abstracted	Not stated	175 Sex trafficked girls and women *Purposive sampling*	9–30	X	X	X	65.9%	Not included
Silverman et al. (2011) [61]	Childhood	Cross-sectional	ASHA Center	Self-administered questionnaire	Not stated	211 HIV infected FSW’s *Purposive sampling*	>18			X	55.70%	Included
Shahmanesh et al.(2009a) ^2^	Childhood	Cross-sectional	Community	Administered questionnaire	Not stated	326 FSW’s *Respondent driven sampling*	>18	*Not specified*: *“sexual abuse”*			4.60%	Not included
Shahmanesh et al.(2009b) ^2^	Childhood	Cross-sectional	Community	Administered structured interview	Not stated	326 FSW’s *Respondent driven sampling*	>18	*Not specified*: *“sexual abuse”*			4.60%	Not included
Bhat et al. (2012) [52]	Childhood	Cross-sectional	Observation home	Self-administered questionnaire	Finkelhor's sexual abuse scale	119 Runaway boys *Purposive sampling*	11–18	X	X	X	35.0%	Included
Deb et al. (2009)^3^ [55]	Childhood	Case Control (120 sexually abused girls and 120 non- sexually abused girls)	Observation home/shelter home and schools	Administered structured interview	Sexual Abuse Screening Questionnaire	240 Young girls *Purposive sampling*	13–18	X	X	X	17%-46%	Included
Banerjee et al. (2008) [51]	Childhood	Cross Sectional	Community	Personal interviews	Not stated	330 Domestic child laborers *Stratified random sampling*	8–14	*Not specified*: *“sexual abuse”*			**3%**	Not included

# Data based on an Intervention Study.

* Mixed Methods Study.

**International Society for the Prevention of Child Sexual Abuse and Neglect Tool-Retrospective (ISPCAN).

^2^ Studies based on same data set but reporting different health outcomes.

^3^ Studies based on same data set but reporting different health outcome.

**Table 7:** Characteristics of quantitative and mixed-method studies included in the review with populations at risk.

**Author**	Period of prevalence	Study design	Setting	Method of data collection	Instruments/ Tools used to measure CSA	Sample size and sampling strategy	Age (in years)	Type of sexual abuse			Prevalence	Perpetration
								Non-contact	Contact	Forced intercourse		
Deb et al. (2011a) #3 [66]	Childhood	Case Control (120 sexually abused girls and 120 non- sexually abused girls)	Observation home/shelter home and schools	Administered structured interview	Sexual Abuse Screening Questionnaire	240 Young girls *Purposive sampling*	13–18	X	X	X	17%-46%	Not included
Deb et al. (2011b) #3 [65]	Childhood	Case Control (120 sexually abused girls and 120 non- sexually abused girls)	Observation home/shelter home and schools	Administered structured interview	Sexual Abuse Screening Questionnaire	240 Young girls *Purposive sampling*	13–18	X	X	X	17%-46%	Not included
Reed et al. (2013) [98]	Childhood	Cross-sectional	Community	Administered structured interview	Not stated	850 FSW’s *Respondent driven sampling*	18–40	X			20.2%	Not included
Jangam et al. (2015) [36]	Childhood	Case control (609 Women with psychiatric disorders as cases and 100 education and age-matched controls)	Clinical facility	Administered structured interview	ISPCAN** Child Abuse Screening Tool-Retrospective (ICAST-R)	709 women *Consecutive sampling method*	18–50	X	X	X	13.3%- Cases 8.0%- Controls	Not reported
Silverman et al. (2014) [73]	Past year experience of CSA	Cross Sectional	ASHA Center	Administered structured interview	Not stated	211 HIV infected FSW’s *Stratified random sampling*	>18		X	X	46.20%	Not included

# Data based on an Intervention Study.

* Mixed Methods Study.

**International Society for the Prevention of Child Sexual Abuse and Neglect Tool-Retrospective (ISPCAN).

^2^ Studies based on same data set but reporting different health outcomes.

^3^ Studies based on same data set but reporting different health outcome.

**Table 8:** Characteristics of quantitative and mixed-method studies included in the review with populations at risk.

**Author**	Period of prevalence	Study design	Setting	Method of data collection	Instruments/ Tools used to measure CSA	Sample size and sampling strategy	Age (in years)	Type of sexual abuse			Prevalence	Perpetration
								Non-contact	Contact	Forced intercourse		
Gaidhane et al. (2008) [30]	Childhood	Cross-sectional	Health camp	Administered structured interview	Non-Standardized	163 Adolescent street boys *Simple random sampling*	11–19			X	32.0%	Not included
Bal et al. (2010) [45]	Past year experience of CSA	Cross sectional	Community	Administered structured interview	Not stated	192 females 362 males and (554 Street children) *Cluster sampling*	11–15	“used for sexual stimulation by any means”			9%	Not included
Deb et al. (2008) * [74]	Childhood	Cross-sectional	Clinical	Administered Semi-structured questionnaire	Not stated	26 FSW’s *Incidental* *Sampling*	<18 to >33			X	Not given	Not included
Devine et al. (2010) * [38]	Childhood	Cross-sectional	Community	Administered structured interviews	Not stated	220 FSW´s *Purposive sampling*	≥18			X	29.6%-44.4%	Not included
Tomori et al. (2016) * [31]	Childhood	Cross-sectional	Community	Administered structured interview	Not stated	11,788 Men who have sex with Men (MSM) *Respondent driven sampling*	≥18 years		X	X	22.4%	Included

# Data based on an Intervention Study.

* Mixed Methods Study

**International Society for the Prevention of Child Sexual Abuse and Neglect Tool-Retrospective (ISPCAN).

^2^ Studies based on same data set but reporting different health outcomes.

^3^ Studies based on same data set but reporting different health outcome

**Table 9:** Summary of qualitative studies and mixed-methods studies included in the review.

Author	Study Design	Methods of Data Collection	Setting	Sample Size and sampling strategy	Age of Population (in years)
Sahay et al. (2008) [58]	Qualitative	FGDS and IDIs	Community	131 Young Men who are Sexual Offenders Tribal Males (85) and Non-Tribal Males (46) *Purposive sampling*	15–26
Karandikar et al. (2013) [40]	Qualitative	Semi-structured Interview	Community	48 Female Commercial Sex Workers *Purposive sampling*	20–60
Rashid et al. (2012) [63]	Qualitative	IDIs	Prison and residence	43 (39 boys and 4 girls) Children in conflict with law *Purposive sampling*	13–18
Basu (2012) [37]	Qualitative	IDIs, participant observations and journal entries	Community	46 Female Commercial Sex Workers *Purposive sampling*	NS
Karandikar et al. (2013) [41]	Qualitative	Semi-structured Interview	Community	10 Commercial Sex Workers *Purposive sampling*	20–33
Magar (2013) ^#^ [42]	Qualitative	FGDs and IDIs	Community	41 Trafficked Girls for Sex Work (Rescued) *Snowball sampling*	12–18
Mimiaga et al. (2015) [35]	Qualitative	IDIs, FGDs and KIs	NGO	55 Men who have Sex with Men *Purposive sampling*	>18
Gupta et al. (2009) [39]	Qualitative	Case-records narratives	NGO	61 Trafficked Girls for Sex Work (survivors) *Purposive sampling*	14–30

**Table 10:** Summary of qualitative studies and mixed-methods studies included in the review.

Author	Study Methodology	Methods of Data Collection	Setting	Sample Size and sampling strategy	Age of Population (in years)
Sahoo et al. (2015) [44]	Qualitative	IDIs	Community	56 females *Purposive sampling*	16–24
Chakrapani et al. (2008) [34]	Qualitative	IDIs	Community	10 Male commercial sex workers (Men who have sex with Men) *Purposive and snowball sampling*	21–52
Sinha (2015) [43]	Qualitative	Participant observation, Life-history interviews, and IDIs	Community	49 Female Commercial Sex Workers *Purposive sampling*	22–50
Sahay et al. (2013) * [59]	Mixed Method	In-depth interview	Schools	130 (110 girls and 20 boys) *Stratified Random sampling*	8–30
Sahay (2010) * [62]	Mixed Method	In-depth interview	Community	350 women (tribal and non-tribal) *Purposive sampling*	10–18
Deb et al. (2008) * [74]	Mixed Method	In-depth Interview and Case Study	Clinical	26 Female Commercial Sex Workers *Incidental sampling*	18–33
Devine et al. (2010) * [38]	Mixed Method	In-depth interview	Community	220 Female Commercial Sex Workers *Purposive sampling*	≥18
Tomori et al. (2016) * [31]	Mixed Method	In-depth Interview and Focus Group Discussion	Community	11,788 Men who have sex with Men *Respondent driven sampling*	≥18 years

Among quantitative studies we extracted three methodological moderators: (a) design of study (primarily cross-sectional, case-control, or otherwise); (b) sampling strategy (random, purposive, or convenience sampling); (c) measurement instrument (name, whether standardised, number of items, etc.). We also extracted the data on the time frame for which CSA was measured (childhood experience or past 12-months experience) for quantitative studies. Three categories of CSA were used: (a) *non-contact sexual abuse* (e.g., exhibitionism, indecent exposure, sexual harassment or voyeurism); (b) *contact sexual abuse without penetration* (e.g., non-genital fondling, kissing, or genital touching); and (c) *forced intercourse* (i.e. anal, oral, or vaginal intercourse), as previously classified in a WHO publication [24]. Depending on the information available for each study, we report prevalence on the total sample or separately on boys and girls.

A synthesis of the qualitative evidence, guided by the review questions, was conducted across the qualitative and mixed-methods studies that are included in the review. The synthesis was performed using an interpretive perspective in which themes were identified in the original papers, compared across studies, and then combined into a whole via a listing of descriptive themes in line with the determinants and perpetration of CSA informed by the socio-ecological framework [25], and the impact of CSA on the study participants (S1 File). The analysis included coding of text 'line-by-line' as a first step that was conducted by one of the authors (RD). The coding was followed by the development of descriptive themes by two authors (VC and RD) independently, followed by discussions to reach consensus. The categorisation of the respective descriptive themes under the individual, family, community, and societal factors was finalised after discussions among the authors (RD, VC, and AKK). The majority of the qualitative and mixed-methods studies focused on high risk populations such as Men Having Sex with Men (MSMs) and Commercial Sex Workers (CSWs) including assessing the HIV risk among these populations, identifying potential pathways for sex workers or trafficked girls to enter sex work, and experiences of abuse and neglect during childhood among such populations. There were only a few papers that exclusively focused on CSA experiences of the participants. In order to account for this, our study coded the relevant sections of each study that focused on CSA experience of the participants.

A separate quality assessment tool for quantitative and qualitative studies was developed for this review. The quality assessment tool for quantitative studies was adapted from Effective Public Health Practice Project (EPHPP) Quality Assessment Tool [26]. The quality assessment tool for qualitative studies was adopted from Consolidated criteria for Reporting Qualitative Research (COREQ) guidelines [27]. Each article was assessed using the quality assessment tool and then all articles were summarized together (Tables 11–14) to give an impression of the overall quality of the studies included in the review.

**Table 11:** Quality assessments of quantitative and mixed-method studies.

	Study References												
EPHPP Item	Charak et al. (2015)	Charak et al. (2014)	Sahay (2013)	Miller et al. (2014)	Hasnain et al. (2006)	Das et al. (2014)	Zolotor et al. (2009)	Deb et al. (2012)	Deb et al. (2010)	Jaisoorya et al. (2015)	Krishnakumar et al. (2014)	Bhilwar et al. (2015)	Jaya et al. (2007)
Domain 1- Selection Bias													
Were the individuals selected to participate in the study likely to be representative of the target population?	×	×	×	×	×	×	×	×	×	√	√	×	√
Was any information regarding non-participation in the study included?	×	×	×	√	×	×	×	×	×	√	×	×	√
Domain- 2 Study Design													
Was there a clear statement of aims/objectives of the study?	√	√	√	√	√	√	√	√	√	√	√	√	√
Is the research design appropriate for the objective?	√	√	√	√	×	√	√	√	√	√	√	√	√
Domain 3- Sampling													
Was the sampling strategy appropriate to the objective?	√	√	√	√	×	√	×	×	×	√	√	×	×
Is the sample size adequate for the objective?	√	√	×	×	×´	√	×	√	×	√	√	√	×
Domain 4- Data Collection tools													
Was the Data collection tool reliable?	√	√	×	√	×	√	√	√	√	√	√	√	×
Was the data collection tool valid?	√	√	×	√	×	√	√	√	√	√	√	√	×
Domain 5- Data Analysis													
Is the statistical analysis appropriate for the study design?	√	√	√	√	×	√	√	√	√	√	×	√	√
Has the confidence intervals or standard errors been Reported?	×	×	√	√	×	√	×	√	√	√	×	×	√
Were the various confounders included and adjusted in the final analysis?	√	√	×	√	×	√	×	×	×	√	×	×	×
Domain 6- Bias													
Has any other bias been reported? What were those biases?	√ (IB; SB)	√ (IB; SB)	×	√ (SB)	×	√ (IB; SB)	√ (IB; SB)	√ (IB; SB)	×	√ (IB; SB)	×	√ (IB; SB)	√ (IB; SB)
Have ethical issues been taken into consideration?	√	√	√	×	×	√	√	√	√	√	×	√	√

EPHPP Effective Public Health Practice Project.

√ Yes.

× No (also includes information not reported).

NA Not Applicable.

IB Information Bias (Includes Observer Bias, Social Desirability, Recall Bias).

SB Selection Bias.

**Table 12:** Quality assessments of quantitative and mixed-method studies.

	Study References												
EHPP Item	Nayak et al. (2010)	Dunne et al. (2009)	Pillai et al. (2008)	Bhattacharya et al. (2012)	Kar et al. (2007)	Sahay et al. (2010)	Sahay et al. (2013)	Pillai et al. (2009)	Balaji et al. (2011)	Silverman et al. (2007)	Silverman et al. (2006)	Silverman et al. (2011)	Shahmanesh et al. (2009a)
Domain 1- Selection Bias													
Were the individuals selected to participate in the study likely to be representative of the target population?	√	×	√	×	×	×	×	×	×	×	×	×	√
Was any information regarding non-participation in the study included?	×	×	√	NA	√	×	×	×	×	√	√	√	√
Domain- 2 Study Design													
Was there a clear statement of aims/objectives of the study?	√	√	√	×	√	√	√	√	√	√	√	√	√
Is the research design appropriate for the objective?	√	√	√	√	√	√	√	√	√	√	√	√	√
Domain 3- Sampling													
Was the sampling strategy appropriate to the objective?	√	×	√	√	×	×	√	√	√	√	√	√	√
Is the sample size adequate for the objective?	×	√	√	√	×	×	×	×	×	×	×	√	√
Domain 4- Data Collection tools													
Was the Data collection tool reliable?	√	√	×	NA	×	×	×	×	×	×	×	×	×
Was the data collection tool valid?	√	√	×	NA	×	×	×	×	×	×	×	×	×
Domain 5- Data Analysis													
Is the statistical analysis appropriate for the study design?	√	√	√	NA	√	×	×	√	√	√	√	√	√
Has the confidence intervals or standard errors been Reported?	√	×	√	NA	×	×	×	√	√	√	√	√	√
Were the various confounders included and adjusted in the final analysis?	√	NA	√	NA	×	×	×	√	√	√	√	√	√
Domain 6- Bias													
Has any other bias been reported? What were those biases?	√(IB; SB)	√(IB; SB)	√(IB; SB)	×	√(IB; SB)	×	×	√(IB; SB)	√(IB; SB)	√ (IB; SB)	√ (IB; SB)	√ (IB; SB)	√ (IB; SB)
Have ethical issues been taken into consideration?	×	√	√	√	√	×	√	√	√	√	√	√	√

EPHPP Effective Public Health Practice Project.

√ Yes.

× No (also includes information not reported).

NA Not Applicable.

IB Information Bias (Includes Observer Bias, Social Desirability, Recall Bias).

SB Selection Bias.

**Table 13:** Quality assessments of quantitative and mixed-method studies.

	Study References													
EHPP Item	Shahmanesh et al. (2009b)	Bhat et al. (2012)	Deb et al. (2009)	Banerjee et al. (2008)	Deb et al. (2011a)	Deb et al. (2011b)	Reed et al. (2013)	Jangam et al. (2015)	Silverman et al. (2014)	Gaidhane et al. (2008)	Bal et al. (2010)	Deb et al. (2008)	Devine et al. (2010)	Tomori et al. (2016)
Domain 1- Selection Bias														
Were the individuals selected to participate in the study likely to be representative of the target population?	√	√	×	×	×	×	√	√	×	×	√	×	×	√
Was any information regarding non-participation in the study included?	√	√	×	×	×	×	×	×	√	√	×	×	×	√
Domain- 2 Study Design														
Was there a clear statement of aims/objectives of the study?	√	√	√	×	√	√	√	√	√	√	×	√	√	√
Is the research design appropriate for the objective?	√	√	√	√	√	√	√	√	√	√	√	√	√	√
Domain 3- Sampling														
Was the sampling strategy appropriate to the objective?	√	√	√	×	√	√	√	√	√	√	√	×	√	√
Is the sample size adequate for the objective?	√	×	×	×	×	×	√	√	×	×	√	×	×	√
Domain 4- Data Collection tools														
Was the Data collection tool reliable?	×	√	×	×	×	×	×	√	×	×	×	×	×	×
Was the data collection tool valid?	×	√	×	×	×	×	×	√	×	×	×	×	×	×
Domain 5- Data Analysis														
Is the statistical analysis appropriate for the study design?	√	√	×	×	×	×	√	√	√	√	√	×	×	√
Has the confidence intervals or standard errors been Reported?	√	√	×	×	×	×	√	√	√	√	√	×	×	√
Were the various confounders included and adjusted in the final analysis?	√	√	×	×	×	×	√	√	√	√	√	×	×	√
Domain 6- Bias														
Has any other bias been reported? What were those biases?	√ (IB; SB)	√ (IB; SB)	×	×	√ (IB; SB)	√ (IB; SB)	√ (IB)	√ (IB; SB)	√ (IB; SB)	√ (IB; SB)	×	√(IB; SB)	√ (IB)	√ (IB; SB)
Have ethical issues been taken into consideration?	√	√	√	×	√	√	√	√	√	√	×	√	√	√

EPHPP Effective Public Health Practice Project.

√ Yes.

× No (also includes information not reported).

NA Not Applicable.

IB Information Bias (Includes Observer Bias, Social Desirability, Recall Bias).

SB Selection Bias.

**Table 14:** Comprehensiveness of reporting of qualitative and mixed-methods studies.

COREQ Item	Sahay et al. (2008)	Karandikar et al. (2011)	Rashid et al. (2012)	Basu (2012)	Karandikar et al. (2013)	Magar (2013)	Mimiaga et al. (2015)	Gupta et al. (2009)	Sahoo et al. (2015)	Chakrapani et al. (2008)	Sinha (2015)	Sahay et al. (2013)	Sahay (2010)	Deb et al. (2008)	Devine et al. (2010)	Tomori et al. (2016)
Domain 1- Research team and Reflexivity																
Interviewer/facilitator identified	×	√	×	√	√	√	√	√	√	√	√	√	√	×	√	√
Researcher credentials	×	×	×	√	√	×	√	×	√	√	√	√	×	×	√	√
Occupation of researcher	×	×	×	√	√	×	√	×	√	√	√	√	×	×	√	√
Gender of researcher	×	×	×	√	√	×	×	×	√	×	√	√	×	×	√	×
Experience and training	×	√	×	√	√	×	√	×	√	×	×	√	×	×	√	√
Prior/existing relationship with participants	×	×	×	√	×	×	×	×	×	×	√	×	×	×	√	√
Participant knowledge of interviewer	×	√	×	√	×	×	×	×	×	×	√	×	×	×	×	√
Interviewer characteristics	×	×	×	√	×	×	√	×	×	√	√	×	×	×	√	√
Domain 2: Study design																
Methodology and theory	×	×	×	√	×	×	√	×	√	√	√	√	√	×	√	√
Sampling strategy	×	√	×	×	×	√	×	√	√	√	√	√	√	√	√	√
Method of approach/invitation	×	√	×	×	√	×	×	×	√	√	√	√	√	√	√	√
Sample size	√	√	√	√	√	√	√	√	√	√	√	√	√	×	√	√
Non-participation	×	×	×	×	×	×	×	√	×	×	×	√	√	×	√	×
Setting of data collection	×	√	√	√	√	√	×	√	×	×	√	√	√	×	√	√
Presence of non-participants	×	×	×	×	×	×	×	×	×	×	×	×	√	×	×	×
Description of sample, for example, demographics	√	√	√	√	√	√	√	√	√	√	√	√	√	√	√	√
Interview guide	×	√	×	×	√	×	√	×	√	√	√	×	×	×	√	√
Audio/visual recording	×	×	×	√	√	×	√	√	√	√	√	×	×	×	√	×
Field notes	×	√	×	√	√	×	×	√	×	×	×	×	×	×	×	×
Duration	×	√	×	√	√	×	√	×	√	√	√	√	√	×	×	×
Data saturation	×	√	×	×	√	×	×	√	×	×	×	×	×	×	×	×
Transcriptions returned	×	×	×	×	×	×	×	×	×	√	√	×	×	×	×	×
Domain 3: Analysis and Findings																
Number of data coders	×	×	×	√	×	×	√	×	√	√	×	×	×	×	√	√
Description of coding tree	×	×	×	√	×	×	√	×		×	×	×	×	×	×	×
Derivation of themes—in advance or derived	×	√	×	√	√	×	√	√	√	√	√	×	×	×	√	√
Software	×	×	×	×	×	×	√	×	√	√	√	×	×	×	√	√
Participant checking	×	×	×	×	×	×	√	×	×	√	√	×	×	×	×	×
Quotations presented	√	√	√	√	√	√	√	√	√	√	√	√	√	×	√	√
Data and findings consistent	√	√	√	√	√	√	√	√	√	√	√	√	√	×	√	√
Clarity of major themes	√	√	√	√	√	√	√	√	√	√	√	√	√	×	√	√
Clarity of minor themes	×	×	×	×	×	×	×	×	×	×	×	×	×	×	×	√
Have ethical issues been taken into consideration	√	√	×	×	√	×	√	√	√	√	×	×	×	×	√	√

COREQ Consolidated criteria for reporting qualitative research.

√ Reported.

× Not Reported.

## Results

### Article yield of systematic search

**Fig 1:**
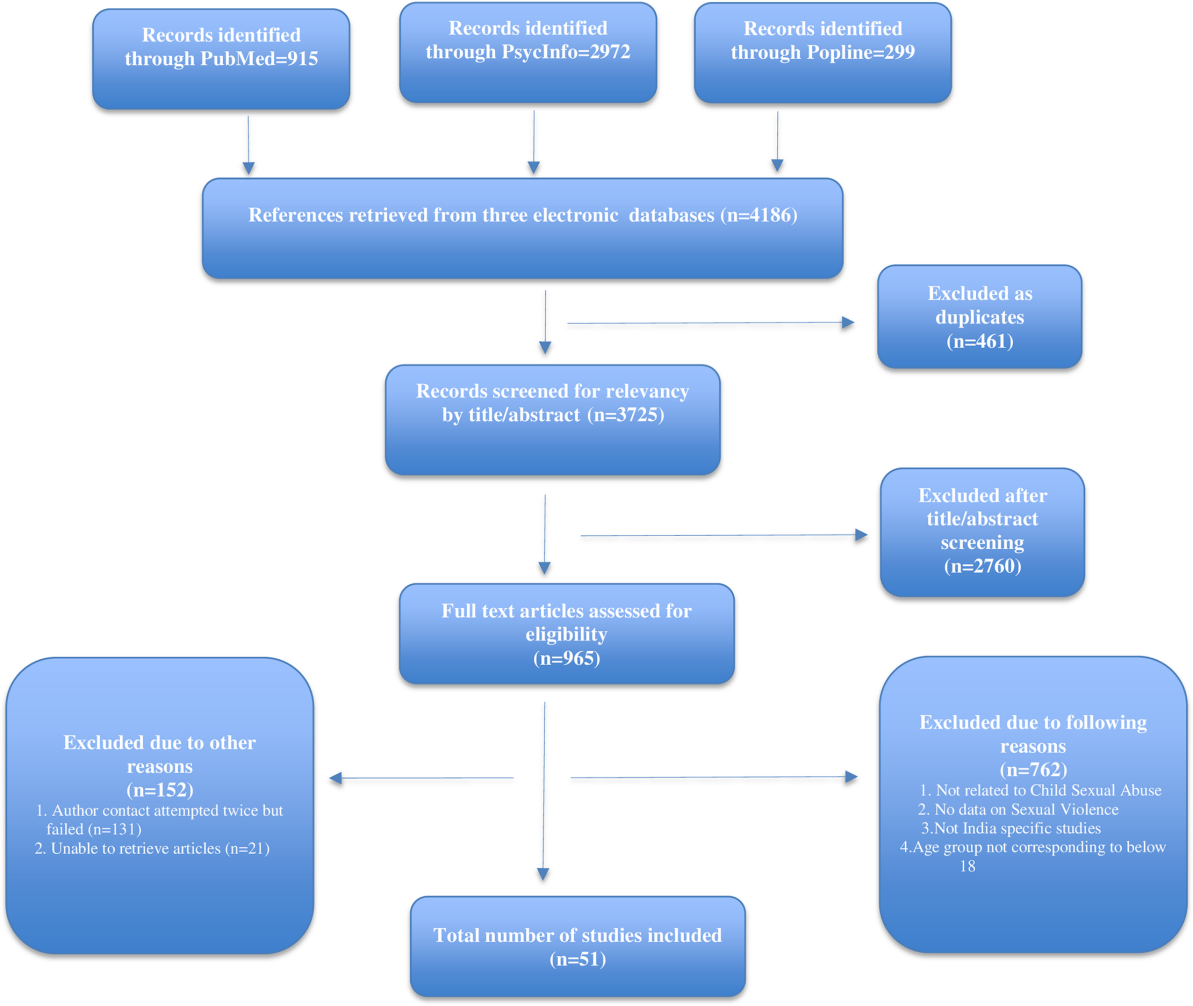
Adapted PRISMA flow chart demonstrating study selection and filter results.

Fig 1 illustrates the flow chart of articles. The initial search of CSA articles published in electronic literature databases (PubMed, POPLINE, and PsycINFO) identified 4,186 potentially eligible studies based on the inclusion criteria. After removal of duplicates, we were left with 3,725 potentially relevant studies that were screened for title and abstract relevancy. After title and abstract screening, 2,760 studies were excluded, leaving 965 for full-text screening applying the same inclusion criteria. Of the 965 articles, 762 articles were excluded because they (1) focused on extraneous topics, (2) lacked Indian context, (3) surveyed a time frame of exposure to sexual abuse for study participants after they attained 18 years of age, or (4) did not collect or analyze original data on CSA. We were unable to retrieve 21 articles through searching our institutional libraries or through contacting the authors (S2 File). Additionally, 131 more articles were excluded from the final analysis, as the articles provided insufficient information to assess eligibility for inclusion and two attempts to contact authors for additional information failed. The final list of included articles consists of 51 studies.

### Study designs and study populations

Among the 51 studies included in the review, 35 studies were based purely on quantitative methods, 11 studies were based purely on qualitative methods while five studies utilized mixed-methods. Most quantitative studies were conducted among respondents from general population (Tables 2–5) while a few of them were conducted among populations at risk (Tables 6–8). The majority of quantitative studies utilized a cross-sectional design with only a few using other designs like case-control or utilizing the case records from medical case histories.

A quarter (13/51) of studies that were included were based in educational institutions. A fifth of the studies (10/51) included community samples of young men and girls while a few other studies (5/51) included women or young adolescents attending gynecology, mental health facilities, or health camps organized by non-governmental organizations (NGOs). Approximately half of the other studies (24/51) were conducted in specific populations such as commercial sex workers, children in shelter homes, sex-trafficked young girls in observational homes, adolescent street boys, runaway children, men who have sex with men (MSM) and children in conflict with law. Most qualitative studies were conducted with specific populations as discussed above.

Almost half the studies included in this review evaluated CSA through interviews with children below 18 years of age (25/51) while the rest of the studies evaluated retrospective recall of CSA experiences among adults. Twenty-one studies were exclusive with girls or women with 17 of these studies among female sex workers and trafficked girls. Nine studies were conducted exclusively with boys including runaway or adolescent boys living on the streets and MSMs. The rest of the studies (20/51) included both boys and girls either from the educational institutions, community samples, health camps, or children in conflict with law.

### CSA definitions and measurement

Around 50% of the studies that utilized purely quantitative methodology, assessed all forms of CSA (forced intercourse, contact, and non-contact). Most of these studies were conducted among students in educational institutions or community populations. All the studies that utilized mixed-methods approach and seven studies among purely quantitative studies used a narrower definition (forced intercourse or rape) of CSA and majority of these studies were conducted among female sex workers. While most of these studies (36/40) evaluated childhood experience of CSA while four studies included the question on experience of CSA in the past year.

Among the 37 studies using quantitative methods, only 16 studies reported use of standardized tools. The various tools that were used were 1. ISPCAN Child Abuse Screening Tool (ICSAT)–C and ICSAT- R, 2. Childhood Trauma Questionnaire, 4. Adapted Questionnaire from MoWCD study, 5. Finkelhor’s Sexual Abuse Scale (used in community studies), and 6. Sexual Abuse Screening Tool. Two studies were based on validation of ICSAT-C and ICSAT-R for measurement of CSA in six countries including India [28, 29].

### Prevalence estimates

The prevalence of CSA ranged from 4%- 41% in studies conducted exclusively among young women below 18 years of age and who are current students while the studies reported a lifetime CSA prevalence of 3–39% among women above 18 years of age. There was a much wider range of CSA prevalence (4%- 57%) reported among boys in educational institutions. One third of the study sample of adolescent street boys reported forced intercourse [30], while almost a quarter of the study sample of men who have sex with men (MSMs) reported experiencing contact sexual abuse with or without forced penetration during childhood [31]. The studies also reported variations in prevalence estimates when they included all forms of CSA (contact, non-contact, and forced intercourse). For example, 35% prevalence of any form of CSA was reported in the age group 15–19 in one study [32] as opposed to 4% among young girls in age range 12 years to 16 years when CSA was specified as “*sexual abuse”* in general [33]. The prevalence estimates of CSA experiences reported among select populations like sex trafficked girls and women ranged from 4% to 66%.

### Determinants of CSA

**Fig 2:**
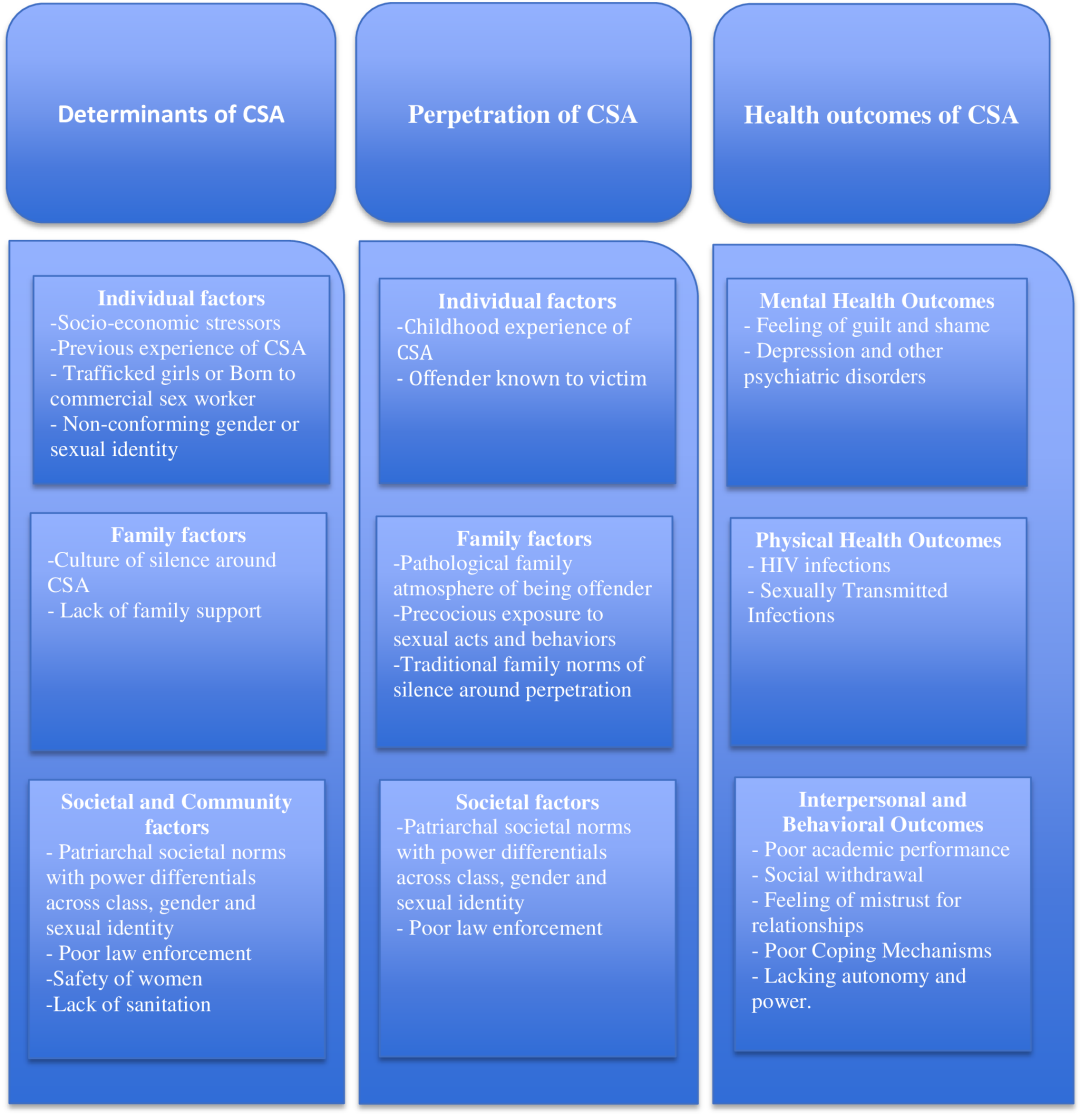
Synthesis of qualitative findings guided by social-ecological framework.

The social-ecological model guided the emergence of *determinants of CSA* as one of the themes in the synthesis of qualitative data across studies included in this review as shown in Fig 2. The synthesis suggests that CSA is a multifaceted phenomenon grounded in the interplay between individual, family, community, and societal factors. The patriarchal societal norms and power differentials in such societies based on class, gender, and sexual preferences emerged as common descriptive themes that increased the risks of CSA across the qualitative studies on CSWs and MSMs [31, 34, 35]. Individual factors like poor socio-economic status, death of a parent or husband, and being born to a commercial sex worker were descriptive themes that emerged as pathways to be initiated in commercial sex work and resultant CSA experiences for minor girls that had been trafficked. Early childhood experience of CSA was also documented as a risk factor for re-victimization as well as initiation into commercial sex work. Lack of proper family support, family and personal history of mental health pathology, and pathological family exposures to sexual images were some of the other potential risk factors, that emerged in the review [36] [37–43]. Lack of sanitation and poor safety of women were also found to be community level factors that increased the risks for CSA from the review of qualitative studies [44]. There were conflicting results in the review of quantitative studies regarding age, gender, family structure (joint vs. nuclear family), and monthly family income as covariates of CSA [30, 45–49]. However negative perception about parents, lower education of mother, and perceived congeniality of family were found to be significantly associated with CSA experience [50]. Domestic child laborers were also found to be at higher risk of all forms of abuse including CSA in one study [51].

### Perpetration of CSA

Few studies (11/51) among the quantitative and mixed-methods studies included in the review reported about the characteristics of the CSA perpetrators [31, 49, 52–61]. The studies conducted among the community sample indicated that perpetrators of CSA in India are known to the abused children, and many of them are family members [49, 58, 59, 62]. The qualitative synthesis of studies that included perpetration as a sphere of enquiry suggests that multiple factors at individual, family, and societal levels play a significant role in perpetration of CSA. The offenders, often known to the victims, take advantage of their accessibility to potential victims and with lack of severe punishment by family members and protective nature of the family members towards the abuser, often leads to the incident getting unreported [58, 59, 62]. However, studies that included adolescent boys as samples reported higher percentage of perpetration by strangers as compared to adolescent girls [49]. The synthesis of data from qualitative studies conducted among MSMs indicated that these perpetrators may be older boys or other men in power like police [31, 57, 63]. A qualitative study among adolescents in our review indicates pathological family atmosphere with precocious exposures to sexual behaviors and sexual acts, traumatic sexual experiences in childhood, sexual interests and exploration, deprivation and failure in romantic relationships, and young boys who have been coerced into homosexual acts are at increased risk of becoming young sexual offenders [58]. Gender inequitable norms were found to be significantly associated with CSA perpetration in a study on evaluation of an intervention to promote gender equality among adolescent boys [57]. However, we need to exercise caution with drawing conclusions about the determinants of perpetration considering the limited number of studies that evaluated the associations, cross-sectional and qualitative study designs, and small sample size of these studies.

### Health outcomes of CSA in India

The health outcomes of CSA can be grouped into mental health, physical health, behavioral and interpersonal (Fig 2). The studies, both quantitative and qualitative, reported high risks for psychiatric disorders [33] including obsessive compulsive disorders [52], suicidal behaviors [64] and depression [32]. The victims of CSA were also found to have increased risks for temperamental problems, poor social adjustment, lack of trust, and insecure relations with parents [48, 55, 65–68]. Lower academic performance was also associated with reporting CSA in one study [50]. Only one quantitative study evaluated the associations between increased risk of Sexually Transmitted Infections (STI) and CSA [69]. The studies suggest that sexually trafficked women and MSMs involved in commercial sex work and had experienced CSA also report high prevalence and risk behaviors for HIV infection [31, 34, 37–39, 45, 70–74]. However, their HIV status in this study could be an outcome of sex work rather than the experience of CSA itself.

### Interventions for CSA in India

Our review found only five intervention studies [54, 57, 65, 66, 75]. One study examined the 12-month efficacy of *Parivartan* (English: *transformation*), a primary prevention program for young boys that aims to prevent perpetration of gender-based violence through promotion of gender equitable norms [57]. The program resulted in statistically significant increase in gender-equitable attitudes but no change in sexual abuse perpetration among the boys who participated in the intervention. Two studies based on the same dataset that included 120 abused girls as cases and 120 non-abused girls as matched comparisons focused primarily on positive effects of general counseling on symptom reduction for CSA survivors in rehabilitation homes set up by the government or an NGO [65, 66]. An exploratory controlled evaluation of a multi component intervention, Yuva Mitr (friend of youth), involving educational institution-based peer and teacher training, and information material to the youth led to decrease in prevalence of sexual abuse among urban youth at end of 18 months as compared to baseline data [75].

### Quality assessment of the published studies

Of all included quantitative studies, approximately half the studies had high risks of selection bias with poor sampling strategy or sample sizes (Tables 11–13). Half of the quantitative studies rated poorly for representativeness of the sample, whereas the same number of studies did not use an acceptable case definition. Among all quantitative studies only ten studies reported risk of any other bias including bias due to social desirability responses, observer bias, or recall bias. Only a quarter of the included studies used a validated tool for measurement of CSA. Majority of studies rated poorly on data analytical techniques while none of the studies met all criteria of quality assessment. Limited information on prevalence estimates for each type of sexual abuse, and heterogeneity of study designs and CSV measures precluded conduct of a meta-analysis.

Among qualitative studies none of the studies included a description of the moderator or the interviewer´s characteristics while half the included studies did not report any theoretical framework in the study design (Table 14). In addition, majority of the studies did not describe the recruitment process for the study participants and their relationships with the research staff. Most of the included studies rated poorly on qualitative data analysis and reporting.

## Discussions

Our systematic review summarizes what is known about the characteristics of CSA and the status of the research on CSA in India during the last decade. It adds to the scant knowledge of CSA and draws attention to the magnitude and severity of the ongoing epidemic in India. The reviewed literature estimates that 4–41% of the girls and 10–55% of the boys in school and college samples have experienced one form (contact, non-contact, forced) of CSA in India. The prevalence figures are much higher among commercial sex workers, street adolescents and children, children working as domestic laborers, MSMs, and women with mental health problems. In addition to highlighting the high frequency of occurrence, the studies in this review begin to highlight the ecological determinants of CSA experience and perpetration along with adverse impact of CSA on social functioning, behavioral issues, mental health, and physical health.

The review highlights the heterogeneity of the methodologies utilized between the included studies. It is difficult to generalize the estimates of CSA in India noted in this review because of the small sample sizes and non-random samples. Further, the different sampling strategies, varying operational definitions of CSA, different study populations (child, adolescent or adult; vulnerable populations, e.g.- street children, children with mental health difficulties etc.), different study settings (in school, colleges, shelter homes, health clinics, or community based), and various instruments for measurement of CSA add to the practical, methodological, and statistical challenges of presenting pooled prevalence estimates, inter-study comparisons, and cross-population comparisons. Our review suggests that the studies that included standardized instruments and comprehensive definitions of CSA (contact, non-contact, and forced intercourse), reported higher prevalence of CSA. A currently available standardized instrument, like ICSAT- C and R, used globally and validated in Indian context could be a tool for methodologically robust measurement across studies for national and international comparisons.

As per the NCRB statistics for 2015, the legislative framework in India- The POCSO Act, 2012 has resulted in increased reporting of CSA [76]. However, the issues related to mandatory reporting of the CSA incidents, lack of clarity of legislation among professionals (medical officers and police), and general lack of professional support for victims of CSA create potential problems for implementation in the Indian context [77]. The socio- cultural beliefs and practices suggested in our qualitative synthesis and others pertaining to parental rights and styles in a closely-knit patriarchal family system, as existing in India, often do not acknowledge that children are individuals with their own rights and often neglect the sexual and other forms of abuse that the child may report [17, 22]. The underreporting of CSA in India can be attributed to the fear of indignity, guilt, denial from the community, associated socio-cultural stigma (especially if the abuse is in the context of the family), not being able to trust government bodies, and a gap in communication between parents and children about this issue [16, 17, 19, 20, 78]. An upcoming paper, based on data extracted for this review, highlights the ethical and measurement issues with respect to training of interviewers on data collection in addition to non-standardized tools for data collection can result in underreporting of CSA in research studies [79]. Another major concern in India is dearth of good monitoring of various juvenile residential institutes. In addition, majority of the healthcare professionals do not have the abilities and are not trained to examine and manage cases of CSA. Hence the few cases that reach these institutions also often go unreported [22].

Our review also exposed gaps in the current understanding of CSA in some populations in India. The findings suggest that young boys in India have similar and sometimes higher prevalence of CSA as girls. This is in accordance with current understanding of CSA in India [16, 17, 19, 20, 80] but the high prevalence of CSA among boys is in contrast with the majority of global trends [4, 6, 81] However, patriarchal society and existing social norms around masculinity and focus on young girls as primary targets for CSA programs leave vulnerabilities of young boys largely unexplored [31].

The studies with at risk populations for CSA, like trafficked girls, also reported substantial variations with higher estimates from studies where the respondents were below 18 years of age and study included all forms of CSA. The few qualitative studies among MSMs and trafficked girls for commercial sex work included in the review suggest early childhood sexual abuse experiences that often reflect power differences between the child and the perpetrator are pathways that lead the victims into commercial sex work. In addition, our review points to increased risks of CSA among certain populations that include children of commercial sex workers, young girls with mental health issues, and adolescent boys and girls out of schools and in labor force (like domestic laborers etc.)

Evidence suggests deviant sexual interests are a major risk factor for CSA [82]. According to research sexual offenders against children can be distinguished into two groups. The first group account for about 60% of officially known offenders and show no sexual preference disorder, but who, for different reasons, sexually abuse children (e.g., sexually inexperienced adolescents seeking a surrogate; persons with poor mental health, or those with antisocial personality disorders, or from traumatizing family constellations). The other groups are those showing a sexual preference disorder, namely pedophilia (erotic preference for prepubescent children) or hebephilia (erotic preference for early pubescent children) who account for about 40% of officially known offenders [83, 84]. A study included in our review that focused on sexual preferences, estimated sexual preferences for children among 3.3% of the respondents (majority of married and women respondents) [56]. We need to exercise caution with drawing conclusions from a single study that was based on small sample size and poor methodology. However, keeping in mind that the prevalence of paedophilia is at least 1% in the male population [85, 86], it is obviously important to do more research in this direction in India, because there are indicators that many paedophiles are reachable before acting out their impulses [87, 88].

While our review yielded a combination of cross-sectional and qualitative studies that provides an insight into the linkages between a few psychological, physical, and behavioral health outcomes and CSA, it also reveals some knowledge gaps and potential research agenda. There is extensive research literature from high-income countries that links any Adverse Childhood Experiences (ACE) like CSA, abuse, neglect, parental violence etc. with poor psychological, social, and physiological outcomes across the lifespan [89]. All ACEs including CSA tend to have a dose-response relationship with many unwanted health and social outcomes including perpetration and victimization of intimate partner violence, sexual re-victimisation, depression, drug abuse, and even mortality [89–92]. Amongst the significant challenges to addressing ACEs including CSA, other forms of abuse, maltreatment, and neglect in India are its huge population of children, poor child welfare service coverage, poverty, gender inequality, and illiteracy. The limited literature in India suggests that CSA does not necessarily occur in isolation and may co-occur with other forms of ACEs in the same child [16, 17, 93]. There is a need to assess the associations between CSA and physical health outcomes like menstrual irregularities; behavioral issues that persist in adult life of CSA survivors including increased risk of perpetration of CSA, increased participation in sex work, re-victimization as adults, high risk sexual behaviors and psychosexual dysfunctions; and delays in developmental milestones leading to deficits in motor, emotional, behavioural, language, psychosocial, social, and cognitive skills among children in Indian context as has been indicated in global literature on consequences of CSA [11, 81]. The qualitative literature [58, 59, 62], included in the review, based on the experiences of sexually abused girls indicated that the reactions of the families to the discovery of CSA often caused re-traumatization and hindered the healing process. More research is needed to understand the complex familial and social factors that influence the wellbeing among victims of CSA to inform programs and policies for prevention and treatment of CSA victims.

The review also highlights the need for research aimed at designing and evaluating programs for primary prevention and treatment of CSA victims. The high prevalence of CSA in India calls for a multi-faceted ecological approach that also includes strategies for impacting policies, laws, and social and cultural norms of patriarchy and gender inequality that surround CSA [16, 17, 94]. There may be potential value in primary prevention approaches, such as adopted in Yuva Mitr (friend of the youth), through multiple components like information dissemination to young people and universal educational programs that could be delivered in schools and aimed at potential victims of all genders, their parents, professionals, and the general public about CSA [75]. In addition, a culturally tailored intervention module with specific adaptation of trauma and abuse-focused Cognitive Behavior Therapy (CBT) could also be developed for an Indian context for victims of CSA [95].

In addition to the limited causal inferences that can be drawn due to near exclusive cross-sectional study designs of most studies, this systematic review suffers some further limitations. Publication bias is a common and well-documented problem in systematic reviews. Despite comprehensive efforts to retrieve all the available data on CSA prevalence rates in India, we might still have failed to identify some non-referenced publications such as reports from civil society organizations that work in the field of CSA in India, other grey literature and literature such as journalistic articles, commentaries, and other reports available in local languages of India. Furthermore, it is likely that the results of this review are biased because not all unpublished data could be accessed. Furthermore, methodological weaknesses of studies limit the reliability and validity of the results. In addition, we included studies whose main aim was to evaluate the CSA experience among Indian children along with studies whose primary objectives may not have been CSA but included CSA as a covariate. However, our goal was not to critically evaluate each individual study, but to comprehensively review the information currently provided in the literature. Despite its limitations, this systematic review makes a significant contribution to research on CSA in India, since it systematically and comprehensively reviewed, structured, and summarized previous research on the prevalence of CSA, and in doing so, provides a future research agenda.

## Conclusions

CSA is a dark reality that is highly prevalent in India and adversely impacts health. Our literature review underscores the need for the development of a standardized definition of CSA and a validated tool for accurate measurement of CSA across India. Moreover, additional in-depth studies of CSA among the general and specific populations like commercial sex workers and MSMs are needed to develop effective ecological models for prevention and treatment of CSA that are sensitive to the diversity of vulnerabilities of children and adolescents in the Indian context. Furthermore, there is definitely a great need for more research concerning the perpetrators of child sexual abuse, including gathering more knowledge about paedophilia in India, in order to enhance primary preventive strategies.

## Supporting information

S1 Checklist PRISMA 2009 checklist for systematic reviews. (DOC)

S1 File Coding sheet for synthesis of qualitative data. (XLSX)

S2 File List of artciles that could not be retrieved. (XLSX)
